# Social media discourse on feminism: A dataset for sentiment analysis in bangla comments

**DOI:** 10.1016/j.dib.2025.112383

**Published:** 2025-12-24

**Authors:** Md. Mijanur Rahman, Md. Sumon Hosen, Zaid Bin Sajid, Fahim Faisal Sifat

**Affiliations:** Department of Computer Science and Engineering, Southeast University, 251/A Tejgaon I/A, Dhaka 1208, Bangladesh

**Keywords:** Bangla abusive language, Natural language processing, Text classification, Hate speech detection

## Abstract

Bangladesh is a socio-culturally diverse country where perspectives on women’s freedom vary significantly. Social media sites are now important places for sharing feminist ideas through public comments and posts. This study offers a detailed collection of 6,830 comments in Bangla about feminism to help analyze public opinion. Most of this data comes from Facebook, with some also from Instagram and Twitter. Data collection involved systematic extraction from public groups and targeted hashtag searches, including  (women’s rights). Native Bangla speakers meticulously annotated each comment by hand to ensure that it was topical and to identify any abusive language. This manual validation procedure guarantees a high-quality dataset appropriate for the study of online gender-based violence in the Bangla language context, sentiment analysis, and abusive language analysis, among other machine learning and NLP tasks. In addition, comments were divided into three sentiment classes: neutral, negative, and positive. This allowed for thorough analysis of feminist discourse on Bangladeshi social media and supervised learning.

Specifications Table

**Table:** 

Subject	Computer Science / Linguistics / Gender Studies
Specific subject area	Natural Language Processing, Hate Speech Detection, Social Media Analysis
Type of data	Text (CSV)
Data collection	Scraped from public posts and comments on Facebook, Instagram, and Twitter
Data source location	Bangladesh
Data accessibility	Repository name: Bengali Abusive Language Based on Feminism Data identification number: DOI: 10.17632/nxnbwwc7bn.2 Direct URL to data: https://data.mendeley.com/datasets/nxnbwwc7bn/2 Instructions for accessing these data: The dataset is publicly available on Mendeley Data. Users can access and download the CSV file directly via the following link: https://data.mendeley.com/datasets/nxnbwwc7bn/2. No registration or special access is required [1].
Related research article	Hate Speech and Offensive Language Detection in Bengali [2]

## 1 Value of the Data

• This data are valuable because this is the first publicly available comprehensive dataset focused on abusive Bangla comments targeting feminism that has been collected manually.Recent research shows that to address online gender based violence, need a variety of annotator opinions for the NLP [3].
• Other researchers can reuse this dataset to train and evaluate abusive language detection models in Bangla, develop hate speech lexicons, study online gender-based abuse, build content moderation tools, and conduct cross-lingual or sociolinguistic research related to feminism and toxic speech [4].
• Facilitates research into online harassment, digital safety, and gender-based violence in social media. Enables the development of automated moderation tools for Bangla content on digital platforms.
• Beneficial for researchers, policymakers, linguists, and AI practitioners working in gender studies and low-resource language processing.

## 2 Background

The motivation for compiling this dataset arose from the increasing prevalence of abusive and hostile comments targeting feminist discourse on Bangla-language social media platforms. Although hate speech and online toxicity have become areas of growing academic interest, available datasets are largely concentrated in English and a few other high-resource languages. Bangla—spoken by over 230 million people primarily in Bangladesh and the Indian state of West Bengal—is the seventh most spoken language globally. The language exhibits several dialects, with the Standard Colloquial Bangla, based mainly on the Kolkata and Dhaka dialects, serving as the predominant form used in formal communication and media. Despite its wide use, Bangla remains underrepresented in this context, especially in relation to annotated resources that capture gender-based abuse [5]. The majority of recent research on the detection of abusive comments in Bangla has concentrated on the classification of cyberbullying using machine learning models and transliterated Bengali-English comments [6,7]. Recent research highlights the importance of detecting abusive language in low-resource languages like Bangla [8]. We have developed this dataset for classifying abusive comments and working with low-recourse languages such as Bangla on the edge of NLP. All of the comments we have manually collected by visiting three popular social media platforms in Bangladesh: Facebook, Instagram, and X. While we collected data, we searched for some hashtags that are related to feminism. Furthermore, three Bangla native speakers reviewed each of data and leveled them to ensure the current annotation and related to feminist discourse to maintain both as they were abusive in nature and relevant to feminist discourse, maintaining both linguistic and contextual accuracy. For the dataset annotation, three individuals involved such as Dr. Md. Masud Shams Aldin (Chairman & Associate Professor), Dr. Hamida Begum (Associate Professor), and Dr. Taha Yeasin (Assistant Professor), all from Southeast University, Bangladesh. Their experience and understanding in Bangla language ensure high accuracy. If this data is part of a research publication, it will give more information about how the dataset was made, how it was collected, and how it was annotated. This background facilitates reproducibility and methodological transitions.

## 3 Data Description

The dataset consists of 6,830 feminism-related Bangla-language comments that were gathered from public posts, pages, and groups on three well-known social media sites: Facebook, Instagram, and Twitter. The collection of user-generated content that represents public opinion on feminist issues in Bangladeshi society was the main goal. We used keyword filtering to find comments that related to feminism context. Hashtags and phrases such as  (women's rights) were employed during data collection. The dataset consists of one UTF-8 encoded file called all_data.csv and is kept in a single folder. This file includes user comments that were gathered from social media platforms and were initially written in both Bangla and English. To keep the dataset consistent, all English comments were translated into Bangla using Google Translate. Each comment was then manually reviewed and labeled with one of three sentiments: Positive, Negative, or Neutral. The all_data.csv file has two columns—one for the Bangla comment and another for its corresponding sentiment label. The dataset does not include any personal details such as usernames, user IDs, or timestamps. A sample of the labeled Bangla comments is shown in Table 1 to give users a clear idea of the dataset format. There are no subfolders or additional files in the repository, making it easy to access and use for tasks such as text classification, sentiment analysis, and other applications involving natural language processing.

**Table 1:** Manual Dataset Annotation.

## 4 Experimental Design, Materials and Methods

**Fig. 1:**
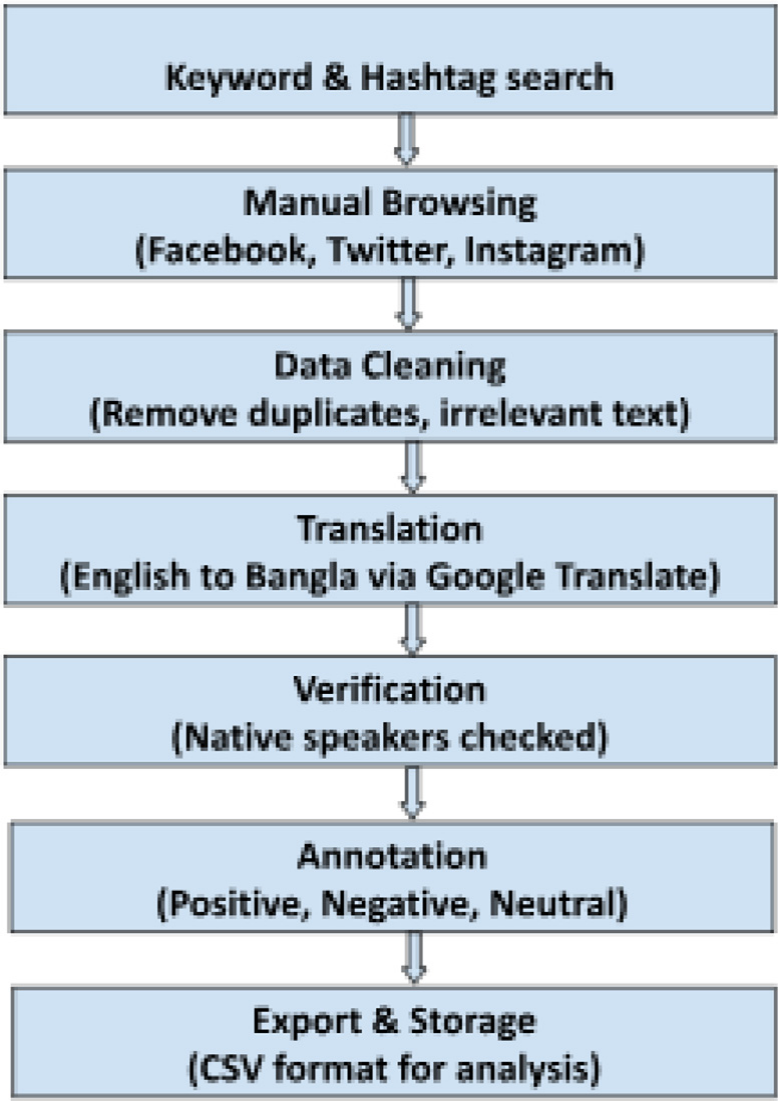
The process of data collection.

**Fig. 2:**
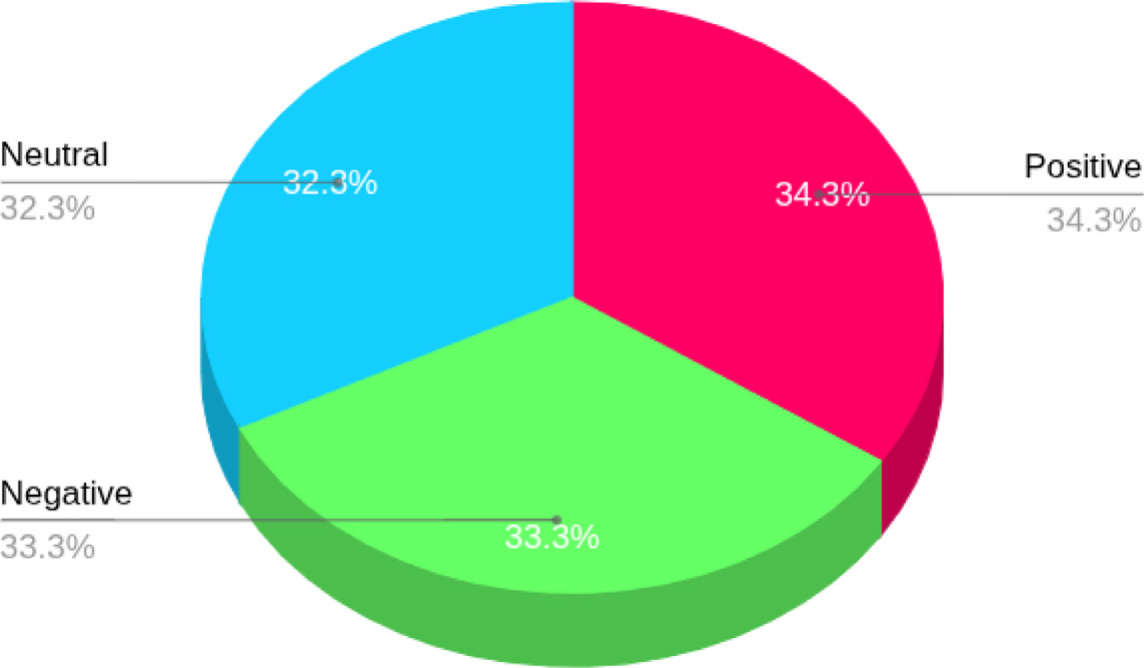
Percentage Distribution.

Fig. 1 shows the data collection process. Between January 2024 and March 2025, we manually collected data from three widely used social media platforms: Facebook, X (formerly Twitter), and Instagram. The goal was to gather Bangla-language comments related to feminism and gender issues. To guide the search, we used a set of common Bangla keywords and hashtags such as  (women’s rights). Across the platforms, these keywords helped us to find relevant posts, comments, and responses. We studied Facebook pages and public groups that support social justice, women's rights, and feminism [4,5]. In a similar manner, we used keyword-based searches to search through threads and replies on Twitter [6]. We searched Instagram captions and comment sections that contained relevant hashtags.All data was gathered manually using a browser; neither automated tools nor scraping software were used. Nonetheless, in order to maintain the natural tone and style of the users' language, we kept original components like hashtags, emojis, and punctuation. Native Bangla speakers then manually annotated each comment with one of three sentiment labels: positive, negative, or neutral. In total, the final dataset includes 6,830 comments, categorized as follows: 2,346 positive, 2,294 negative, and 2,190 neutral. The entire process—from collection to annotation—was carried out using Microsoft Excel for organization and review. Once the annotation was complete, the data was exported as a CSV file for easier use in analysis. Fig. 2 illustrates the sentiment distribution within the dataset.

## Limitations

Although the dataset provides valuable insight into abusive language in Bangla related to feminism, there are still limitations that remain. Firstly, all of the data was collected from Facebook, Twitter, and Instagram, but the data was not collected from other social media platforms such as YouTube and TikTok. Secondly, due to time and access limitations, we might have unintentionally left out some relevant comments or posts because the comments were manually gathered by looking through public posts and groups. The inter-annotator agreement (Kappa) was not computed. However, despite these limitations, the dataset serves as a significant resource for Bangla natural language processing and sentiment analysis related to social issues.

## Ethics Statement

The data used in this study were collected from publicly accessible posts, pages, and groups on Facebook, Instagram, and Twitter. All comments were obtained from open sources where users had shared content publicly. No private, restricted-access, or sensitive information was included. To ensure ethical compliance, all personally identifiable information (PII), such as usernames, profile links, and timestamps, has been fully anonymized. Since the data were publicly available and anonymized, informed consent from individual users was not required. Additionally, the data collection process complied fully with the terms of service and data redistribution policies of the respective social media platforms. No automated scraping tools were used, and the data were not accessed from private profiles or groups. As per our institutional policy, formal ethical approval was not required for this type of anonymized and publicly available data.

## Credit Author Statement

**Md. Mijanur Rahman:** Supervision, Conceptualization, Methodology, Review & Editing; **Md. Sumon Hosen:** Data Collection, Data Curation, Annotation, Formal Analysis, Writing, and Original Draft; **Zaid Bin Sajid:** Data Collection, Annotation, Validation, Visualization, Writing, and Original Draft; **Fahim Faisal Sifat:** Data storing, managing, and converting to CSV format.

## Data Availability

Bengali Abusive Language Based on Feminismall_data.csv (Original data).

## Appendix Supplementary materials

Image, application 1
