# Supplementary material for: Social media discourse on feminism: A dataset for sentiment analysis in bangla comments
Source: Data Brief. 2025 Dec 24;64:112383. doi: 10.1016/j.dib.2025.112383 (PMC12855587; doi:10.1016/j.dib.2025.112383)
Supplement: Supplementary file 1 [file mmc1.pdf]

Data in Brief
Open access
Article template

ARTICLE INFORMATION

Article title

A Resource for Abusive Language Detection in Bangla: Feminism-Related Social Media Comments.

Authors

Md. Mijanur Rahman * Md. Sumon Hosen * Zaid Bin Sajid * Fahim Faisal Sifat

Affiliations

Department of Computer Science and Engineering, Southeast University, Dhaka, Bangladesh, 251/A Tejgaon I/A, Dhaka 1208

Corresponding author's email address and Twitter handle

Email: csesumon8@gmail.com Twitter: https://x.com/csesumon8

Keywords

Bangla abusive language, Natural language processing, Text classification, Hate speech detection.

Abstract:

Bangladesh is a diverse country; there are several people who have distinct views on women's freedom. In social media, individuals often express their views through comments and posts about feminism. To highlight public opinion and support for the researchers, a comprehensive dataset has been developed that contains 6830 public comments in Bangla toward feminism. All the comments have been gradually collected from three social media platforms: Facebook, Instagram, and Twitter. To collect the dataset, we not only visited public groups but also searched based on hashtags: "নারীবাদ" (feminism), "নারীবাদী" (feminist), and "নারীর অধিকার" (women's rights). Moreover, each comment comes under scrutiny by native Bangla speakers to confirm its abusive nature and direct relevance to feminist topics. Due to manual verification, this is a high-quality dataset that would be very effective for machine learning models and natural language processing, such as abusive language detection, sentiment analysis, and online gender-based violence, with particular attention to the Bangla language. Furthermore, in this dataset, each comment has been leveled into one of three categories: positive, negative, and neutral. It provides opportunities for researchers to easily train their models and experiment with social media public opinion based on feminism.

SPECIFICATIONS TABLE

**Subject**	Computer Science / Linguistics / Gender Studies
**Specific subject area**	Natural Language Processing, Hate Speech Detection, Social Media Analysis
**Type of data**	Text (CSV)
**Data collection**	Scraped from public posts and comments on Facebook, Instagram, and Twitter
**Data source location**	Bangladesh
**Data accessibility**	Repository name: Bengali Abusive Language Based on Feminism<br>Data identification number: DOI: 10.17632/nxnbwwc7bn.2<br>Direct URL to data: https://data.mendeley.com/datasets/nxnbwwc7bn/2<br>Instructions for accessing these data: The dataset is publicly available on Mendeley Data. Users can access and download the CSV file directly via the following link: https://data.mendeley.com/datasets/nxnbwwc7bn/2 . No registration or special access is required [1].
**Related research article**	Hate Speech and Offensive Language Detection in Bengali [2]

VALUE OF THE DATA

- These data are valuable because this is the first publicly available comprehensive dataset focused on abusive Bangla comments targeting feminism that has been collected manually.

- Other researchers can reuse this dataset to train and evaluate abusive language detection models in Bangla, develop hate speech lexicons, study online gender-based abuse, build content moderation tools, and conduct cross-lingual or sociolinguistic research related to feminism and toxic speech.

- Facilitates research into online harassment, digital safety, and gender-based violence in social media. Enables the development of automated moderation tools for Bangla content on digital platforms.

- Beneficial for researchers, policymakers, linguists, and AI practitioners working in gender studies and low-resource language processing.

BACKGROUND

The motivation for compiling this dataset arose from the increasing prevalence of abusive and hostile comments targeting feminist discourse on Bangla-language social media platforms. Although hate speech and online toxicity have become areas of growing academic interest, available datasets are largely concentrated in English and a few other high-resource languages. Bangla—spoken by over 230 million people—remains underrepresented in this context, especially in relation to annotated resources that capture gender-based abuse [3]. This dataset was created within the broader scope of natural language processing (NLP), focusing specifically on toxic comment classification and the challenges of working with low-resource languages. Abusive comments were collected from social media platforms by visiting public comments, targeting feminist-related hashtags, and targeting terms commonly used on platforms such as Facebook, Instagram, and X. Moreover, native Bangla speakers manually reviewed and annotated all comments to ensure that they were abusive in nature and relevant to feminist discourse, maintaining both linguistic and contextual accuracy. If this data article accompanies a related research publication, it provides complementary details regarding the dataset's construction, collection criteria, and annotation process. This background enables reproducibility and methodological transparency for those conducting further NLP tasks or linguistic analysis based on the data.

DATA DESCRIPTION

The dataset consists of 6,830 Bangla-language comments related to feminism, collected from public posts, pages, and groups on three popular social media platforms: Facebook, Twitter, and Instagram. The primary objective was to gather user-generated content that reflects public sentiment toward feminist issues in Bangladeshi society. A keyword-based filtering method was applied to extract comments that matched specific terms related to the targeted content. Hashtags and phrases such as "নারীবাদ" (feminism), "নারীবাদী" (feminist), and "নারীর অধিকার" (women's rights) were employed during data collection. The dataset is stored in a single folder and includes one UTF-8 encoded file named all_data.csv. This file contains user comments originally written in both Bangla and English, which were collected from social media platforms. To keep the dataset consistent, all English comments were translated into Bangla using Google Translate. Each comment was then manually reviewed and labeled with one of three sentiments: Positive, Negative, or Neutral. The all_data.csv file has two columns—one for the Bangla comment and another for its corresponding sentiment label. The dataset does not include any personal details such as usernames, user IDs, or timestamps. A sample of the labeled Bangla comments is shown in Table 1 to give users a clear idea of the dataset format. There are no subfolders or additional files in the repository,

making it easy to access and use for tasks like sentiment analysis, text classification, or other natural language processing applications.

SI. NO.	Comment	Label
0	সহবাস কইরা দিলা খালাম্মারে	Negative
1	খুব ভাল লাগল আপু আপনার কথা গুলা শুনে	Positive
2	সাহস থাকলে একদিন মেকআপ ছাড়া সামনে আসো তো?	Negative
3	ভাই মালটাকে নাইট ক্লাবে নিয়ে যান একটু😀	Negative
4	আল্লাহ তা,আলা আমাদের যে হুকুম করেছেন সেইভাবেই ...	Neutral
5	খুব সুন্দর 👍	Positive
6	তার লিখিত বই গুলো তোমার মা মেয়ে ও বোন কে পড়তে দিও	Neutral
7	ওহ। আরেকজন খদ্দের পাওয়া গেল।	Negative
8	মাশা-আল্লাহ, লেখাটা পড়ে ভালো লাগলো।	Positive
9	দেখবে কেউ না কেউ ওদের পক্ষে দাঁড়িয়ে যাবে!আমি...	Neutral

Table 1: Manual Dataset Annotation

EXPERIMENTAL DESIGN, MATERIALS AND METHODS

Between January 2024 and March 2025, we manually collected data from three widely used social media platforms: Facebook, X (formerly Twitter), and Instagram. The goal was to gather Bangla-language comments related to feminism and gender issues. To guide the search, we used a set of common Bangla keywords and hashtags such as “নারীবাদ” (feminism), “নারীবাদী” (feminist), and “নারীর অধিকার” (women’s rights). These keywords helped us locate relevant posts, comments, and replies across the platforms. On Facebook, we explored public groups and pages that focus on feminism, women’s rights, and social justice[4][5]. Similarly, on Twitter, we browsed through threads and replies using keyword-based searches[6]. For Instagram, we looked into captions and comment sections under posts that included relevant hashtags. All data was gathered manually using a browser—no automated tools or scraping software were used. This ensured we complied with platform policies and maintained data quality. After collecting the data, we cleaned the dataset by removing duplicates and excluding non-Bangla content. However, we preserved original elements such as punctuation, emojis, and hashtags to retain the natural style and tone of the users' language. Native Bangla speakers then manually annotated each comment with one of three sentiment labels: positive, negative, or neutral. In total, the final dataset includes 6,830 comments, categorized as follows: 2,346 positive, 2,294 negative,

and 2,190 neutral. The entire process—from collection to annotation—was carried out using Microsoft Excel for organization and review. Once the annotation was complete, the data was exported as a CSV file for easier use in analysis. Figure 1 illustrates the sentiment distribution within the dataset.

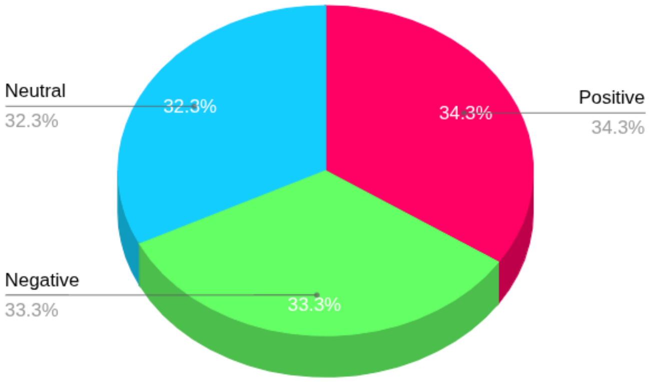

Figure 1: Percentage Distribution

LIMITATIONS

Although the dataset provides valuable insight into abusive language in Bangla related to feminism, there are still limitations that remain. Firstly, all of the data was collected from Facebook, Twitter, and Instagram, but the data was not collected from other social media platforms such as YouTube and TikTok. Secondly, due to time and access limitations, we might have unintentionally left out some relevant comments or posts because the comments were manually gathered by looking through public posts and groups. However, despite these limitations, the dataset serves as a significant resource for Bangla natural language processing and sentiment analysis related to social issues.

ETHICS STATEMENT

The data used in this study were collected from publicly accessible posts, pages, and groups on Facebook, Instagram, and Twitter. All comments were obtained from open sources where users had shared content publicly. No private, restricted-access, or sensitive information was included. To ensure ethical compliance, all personally identifiable information (PII), such as usernames, profile links, and timestamps, has been fully anonymized. Since the data were publicly available and anonymized, informed consent from individual users was not required. Additionally, the data collection process complied fully with the terms of service and data redistribution policies of the respective social media platforms. No automated scraping tools

were used, and the data were not accessed from private profiles or groups. As per our institutional policy, formal ethical approval was not required for this type of anonymized and publicly available data.

CRediT AUTHOR STATEMENT

Md. Mijanur Rahman: Supervision, Conceptualization, Methodology, Review & Editing. Md. Sumon Hosen: Data Collection, Data Curation, Annotation, Formal Analysis, Writing, and Original Draft. Zaid Bin Sajid: Data Collection, Annotation, Validation, Visualization, Writing, and Original Draft. Fahim Faisal Sifat: Data storing, managing, and converting to CSV format.

ACKNOWLEDGEMENTS

The authors would like to thank the individuals who supported the annotation process and provided feedback during data validation. Their contributions were invaluable in ensuring the quality of the dataset.

This research did not receive any specific grant from funding agencies in the public, commercial, or not-for-profit sectors.

DECLARATION OF COMPETING INTERESTS

The authors declare that they have no known competing financial interests or personal relationships that could have appeared to influence the work reported in this paper.

REFERENCES

[1] M. S. Hosen, "Bengali Abusive Language Based on Feminism," vol. 2, July 2025, doi: 10.17632/nxnbwwc7bn.2.

[2] M. Das, S. Banerjee, P. Saha, and A. Mukherjee, "Hate Speech and Offensive Language Detection in Bengali," in *Proceedings of the 2nd Conference of the Asia-Pacific Chapter of the Association for Computational Linguistics and the 12th International Joint Conference on Natural Language Processing (Volume 1: Long Papers)*, Online only: Association for Computational Linguistics, 2022, pp. 286–296. doi: 10.18653/v1/2022.aacl-main.23.

[3] E. A. Emon, S. Rahman, J. Banarjee, A. K. Das, and T. Mittra, "A Deep Learning Approach to Detect Abusive Bengali Text," in *2019 7th International Conference on Smart Computing and Communications, ICSCC 2019*, Institute of Electrical and Electronics Engineers Inc., Jun. 2019. doi: 10.1109/ICSCC.2019.8843606.

[4] “(6) United Nations Development Programme in Bangladesh Reels | Facebook.” Accessed: Jul. 12, 2025. [Online]. Available: https://web.facebook.com/UNDPBD/reels/

[5] “(6) Video | Facebook.” Accessed: Jul. 12, 2025. [Online]. Available: https://web.facebook.com/watch/?ref=search&v=1316948592745483&external_log_id=d728b09b-8270-4679-a7dc-ca5e5c7f0786&q=%23%E0%A6%A8%E0%A6%BE%E0%A6%B0%E0%A7%80%E0%A6%B0%20%E0%A6%85%E0%A6%A7%E0%A6%BF%E0%A6%95%E0%A6%BE%E0%A6%B0%20%23%E0%A6%AE%E0%A6%BF%E0%A6%A5%E0%A6%BF%E0%A6%B2%E0%A6%BE

[6] “#নারীবাদী - Search / X,” X (formerly Twitter). Accessed: Jul. 12, 2025. [Online]. Available: https://x.com/search?src=typed_query&q=%23%E0%A6%A8%E0%A6%BE%E0%A6%B0%E0%A7%80%E0%A6%AC%E0%A6%BE%E0%A6%A6%E0%A7%80
